# Preferential translation of p53 target genes

**DOI:** 10.1080/15476286.2022.2048562

**Published:** 2022-04-07

**Authors:** Miharu Hisaoka, Johanna Schott, Toman Bortecen, Doris Lindner, Jeroen Krijgsveld, Georg Stoecklin

**Affiliations:** a Division of Biochemistry Mannheim Institute for Innate Immunoscience (MI3) and Mannheim Cancer Center (MCC), Medical Faculty Mannheim of Heidelberg University, Mannheim, Germany; b Center for Molecular Biology of Heidelberg University (ZMBH), DKFZ-ZMBHAlliance, Heidelberg, Germany; c National Center for Tumor Diseases (NCT) partner site, Heidelberg, Germany; d Division of Proteomics of Stem Cells and Cancer, German Cancer Research Center (DKFZ), Heidelberg, Germany; e Faculty of Bioscience, Heidelberg University, Heidelberg, Germany; f Medical Faculty, Heidelberg University, Heidelberg, Germany

**Keywords:** P53, translational regulation, isoform specific translation, Ribo-Seq, Sestrin, CDKN1A

## Abstract

The transcription factor p53 exerts its tumour suppressive effect through transcriptional activation of numerous target genes controlling cell cycle arrest, apoptosis, cellular senescence and DNA repair. In addition, there is evidence that p53 influences the translation of specific mRNAs, including translational inhibition of ribosomal protein synthesis and translational activation of MDM2. A challenge in the analysis of translational control is that changes in mRNA abundance exert a kinetic (passive) effect on ribosome densities. In order to separate these passive effects from active regulation of translation efficiency in response to p53 activation, we conducted a comprehensive analysis of translational regulation by comparative analysis of mRNA levels and ribosome densities upon DNA damage induced by neocarzinostatin in wild-type and TP53^−/−^ HCT116 colorectal carcinoma cells. Thereby, we identified a specific group of mRNAs that are preferentially translated in response to p53 activation, many of which correspond to p53 target genes including MDM2, SESN1 and CDKN1A. By subsequent polysome profile analysis of SESN1 and CDKN1A mRNA, we could demonstrate that p53-dependent translational activation relies on a combination of inducing the expression of translationally advantageous isoforms and *trans*-acting mechanisms that further enhance the translation of these mRNAs.

## Introduction

*TP53* is the most frequently mutated gene in human cancer as loss of the p53 tumour suppressor function provides cancer cells with an advantage in cell proliferation and reduced rates of apoptosis [1,2]. p53 is activated by various types of cellular stress including DNA damage, spindle damage, hypoxia or oncogene activation, and directly regulates the transcription of numerous target genes, thereby controlling cell cycle arrest, apoptosis, cellular senescence and DNA repair [2,3]. *CDKN1A* was among the first p53 target genes identified [4], encoding the cyclin-dependent kinase inhibitor p21-CIP1, which is central to p53-dependent cell cycle arrest [5,6]. Another major target of p53 is the proto-oncogene *MDM2*, an E3 ubiquitin ligase that binds to p53 and triggers its proteasomal degradation [7,8].

p53 is further known to interfere with anabolic signalling pathways, e.g. through its transcriptional targets Sestrin 1 (SESN1) and Sestrin 2 (SESN2), which inhibit mTORC1 activity through activation of AMPK [9] and binding to the GATOR2 complex [10,11]. mTORC1 specifically enhances the translation of mRNAs containing 5’ terminal oligopyrimidine (TOP) motifs, which encode ribosomal proteins (RPs) and a range of translation factors [12–14]. Indeed, p53 activation was found to repress the translation of RPs in a SESN1/2-dependent manner [15]. Additional mRNAs were also found to be repressed by p53 at the level of translation including its own mRNA as well as FGF2, CDK4 and MDMX mRNAs [16–17].

The opposite effect, i.e. p53-dependent translational activation, was identified early on for its transcriptional target *MDM2* [20,21]. More recent transcriptome-wide assessments of polysome-associated mRNAs indicate that p53 affects the translation of numerous mRNAs [22,23], including an increase in the translation of several apoptosis regulators [24]. While the underlying mechanisms are largely unknown, a GC-rich *cis*-acting motif as well the RNA helicase DHX30 and the RNA-binding protein (BP) PCBP2 were implicated in controlling the translation of specific mRNAs upon p53 activation [25]. Hence, it is clear that p53 has profound effects on the translation of specific groups of mRNAs [26], yet the extent and molecular mechanisms of translational control by p53 are not well understood.

A major challenge in the analysis of translational control downstream of p53 is the interference with the primary transcriptional response. While state-of-the-art translational analysis by ribosome footprinting (Ribo-Seq) allows calculation of ribosome densities (RD) for individual mRNAs [27], it is important to note that RD is strongly affected by changes in mRNA levels. As we could recently show by measuring the RD of nascent mRNA, newly synthesized mRNAs require time to be fully loaded with ribosomes [28]. This causes a kinetic (passive) distortion whereby the ribosome load of transcriptionally induced mRNAs is transiently reduced, which leads to a negative correlation between changes in ribosome load and changes in mRNA levels [28,29]. As a consequence, the analysis of translational regulation in a dynamic system, where gene expression is not at steady state, requires careful distinction of passive effects on RD from active regulation of translation [28]. Given that p53 causes a strong transcriptional response, we here set out to assess the impact of p53 on the translation of individual mRNAs using Ribo-Seq, taking into account the passive distortion of RD. Thereby, we were able to confirm translational attenuation of 5ʹTOP mRNAs by p53 activation, and identify a group of mRNAs that benefit from active mechanisms providing a translational advantage in a p53-dependent manner.

## Results

### A cellular system to study translational regulation upon p53 activation

**Figure 1.**
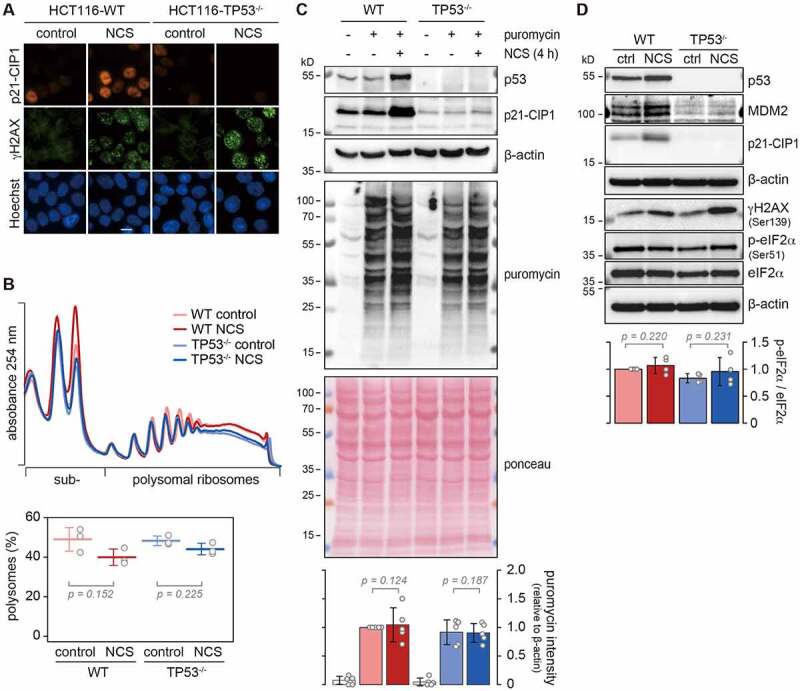
**Assessment of global protein synthesis upon p53 activation**. (A) HCT116-WT and TP53^−/−^ cells were kept under control conditions or treated with NCS (0.2 µg/ml) for 4 h, and subjected to immunofluorescence microscopy using antibodies against p21-CIP1 (red) and Ser139-phosphorylated (γ)H2AX (green); nuclei were stained with Hoechst (blue), scale bar = 10 µm. (B) Polysome profiles were recorded from cells treated as in (A) by sucrose density gradient ultracentrifugation. The percentage of polysomes relative to total ribosomes was calculated by measuring the area under the polysome profile curve (mean ± SD, n = 3, p-values calculated by one-way ANOVA). (C) Cells were cultured under the same conditions as in (A), and puromycin (1 µg/ml) was added to the medium 5 min prior to lysis. Nascent protein production was assessed by Western blot analysis using an anti-puromycin antibody. Specific antibodies were used to detect expression of p53 and p21-CIP1; β-actin and ponceau staining serve as loading controls. Puromycin incorporation was quantified by normalizing the puromycin signal to β-actin (mean ± SD, n = 5, p-values calculated by one-way ANOVA). (D) From cells treated as in (A), protein lysates were prepared for Western blot analysis using antibodies against p53, MDM2, p21-CIP1, γH2AX, Ser51-phosphorylated eIF2α, eIF2α and, as loading control, β-actin. The intensity of phosphorylated eIF2α was normalized to total eIF2α and depicted as a relative value to the WT control condition (mean ± SD, n = 4, p-values were calculated by one-way ANOVA).

As a model system to assess the effect of p53 on protein synthesis, we made use of the human colorectal cancer cell line HCT116 and a mutant counterpart, HCT116-TP53^−/−^, carrying a genomic deletion in the *TP53* gene generated by the Vogelstein lab [30]. In wild-type (WT) HCT116 cells, DNA damage induced by treatment with neocarzinostatin (NCS) causes elevated expression of p53 (Supplementary Figure S1A). As a consequence of p53 activation, the p53 targets p21-CIP1 and MDM2 are induced, reaching their maximum levels at 5 h of NCS treatment (Supplementary Figures S1B and S1C). As expected, p21-CIP1 is not induced in HCT116-TP53^−/−^ cells (Fig. 1 and Supplementary Figure S1B). Fig. 1A shows that NCS was efficient in causing DNA damage in both WT and TP53^−/−^ cells, as visualized by immunofluorescence staining for serine 139-phosphorylated (γ)H2AX. Since we wanted to detect early effects of p53 activation on protein synthesis, we selected 4 h of NCS treatment for all subsequent experiments.

To explore the effect of DNA damage on global protein synthesis, we recorded the distribution of polysomes by sucrose density gradient centrifugation, and observed only a small reduction in polysomal ribosomes upon 4 h NCS treatment, both in WT and TP53^−/−^ HCT116 cells (Fig. 1B). As an alternative method, we used puromycin incorporation assays, which did not show a difference in global protein synthesis upon NCS treatment (Fig. 1C). Likewise, phosphorylation of eIF2α at serine 51, a mechanism by which protein synthesis is repressed under various stress conditions [31], was not altered by NCS treatment or between the two cell lines (Fig. 1D). From these results we concluded that there are no major changes in global protein synthesis under the chosen conditions of DNA damage, and we therefore turned to analysing the effect of p53 activation on the translation of specific mRNAs.

Given that p53 was reported to inhibit the translation of RPs through the induction of its target genes SESN1 and SESN2 in human primary fibroblasts [15], we first validated our cell model by testing the repression of 5ʹTOP mRNAs. To this end, we measured the distribution of two RP mRNAs across polysome profiles by RT-qPCR. The sucrose density gradients were eluted into 13 fractions covering free (#1, 2), subpolysomal (#3–5) and polysomal RNA (#6–13; Supplementary Figure S2A). Indeed, the distribution of both RPL27 and RPL28 mRNAs shifted towards lighter fractions in WT cells upon NCS treatment, but not in TP53^−/−^ cells (Supplementary Figures S2B, S2C, S3A and S3B). In contrast, PRKAB1 mRNA, which serves as a negative control, did not show such a shift (Supplementary Figures S2D and S3C). The degree of polysome association was approximated by calculating the area above the cumulative mRNA distribution. Reduced polysome association upon p53 activation was consistently observed for RPL27 and RPL28 mRNAs in WT cells but not in TP53^−/−^ cells, whereas PRKAB1 mRNA showed barely any change in WT and TP53^−/−^ cells (Supplementary Figures S2B–S2D, panels on the right side). These results demonstrate that RP mRNAs are attenuated at the level of translation in a p53-dependent manner, as reported earlier [15], and validate HCT116 cells as a model suitable for the analysis of p53-dependent translational regulation.

### Transcriptome-wide analysis of DNA damage-induced translational changes

**Figure 2.**
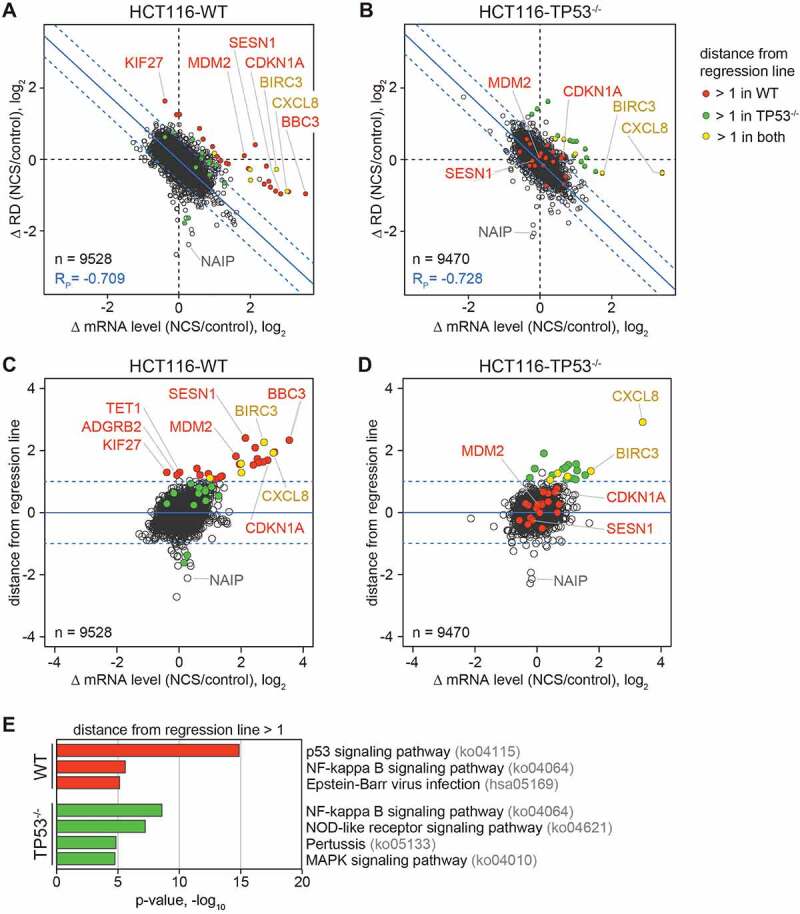
**Transcriptome-wide analysis of translational regulation upon DNA damage**. (A), (B) Ribo-Seq analysis was conducted with (A) HCT116-WT and (B) HCT116-TP53^−/−^ cells under control conditions and after treatment with NCS (0.2 µg/ml) for 4 h, with four biological replicates (n = 4). Read counts were normalized to library size and genes with fewer than 10 reads in the RNA-Seq analysis of NCS-treated cells were excluded from the analysis. Ribosome densities (RD) were calculated by normalizing ribosome footprint values to the input level of the corresponding mRNA. The NCS-induced fold change in RD (log_2_) was plotted against the fold change in mRNA level (log_2_), and the regression line (blue) ± SD (dashed blue) is indicated together with the Pearson correlation coefficient (R_P_). mRNAs with a distance from the regression line > 1 are considered translationally up-regulated, and colour coded according to translational up-regulation in WT cells only (red), in TP53^−/−^ cells only (green) or in both (yellow). (C), (D) The same Ribo-Seq analysis is represented by plotting the orthogonal distance from regression line in (A, B) against the fold change in mRNA level (log_2_). (E) Genes encoding mRNAs found to be translationally up-regulated in WT cells (red) or TP53^−/−^ cells (green) were subjected to pathway and gene ontology analysis using Metascape, and enriched pathways are depicted according to the KEGG database.

We then explored p53-dependent changes in translation by Ribo-Seq analysis, taking into account the kinetic (passive) effect of transcriptional changes on RD. To this end, we compared NCS-induced changes in RD (Δ RD, log_2_) with changes in mRNA levels (Fig. 2A–2D). As expected from our earlier work on the inflammatory response [29], we observed a strong negative correlation between Δ RD and Δ mRNA level in both WT and TP53^−/−^ cells (Fig. 2A and 2B, Pearson correlation coefficient R_P_ = −0.709, and −0.728, respectively), reflecting the kinetic (passive) effect of changes in mRNA levels. This effect is due to the fact that newly synthesized mRNAs require time to be fully loaded with ribosomes. When transcription rates change after treatment with NCS, the proportion of young, not fully loaded mRNA is transiently altered. Therefore, RD measurements are distorted as long as mRNA levels are far from steady state conditions [28]. By taking into account the kinetic effect, our analysis revealed distinct groups of mRNAs whose Δ RD was larger (distance from the regression line > 1) than that of bulk mRNA in WT cells only (red, Fig. 2A), in TP53^−/−^ cells only (green, Fig. 2B), and in both WT and TP53^−/−^ cells (yellow, Fig. 2A and 2B). The same groups were also visualized by calculating the distance from the regression line for each mRNA (Fig. 2C and 2D, Supplementary Table S1, values for all mRNAs in Supplementary Table S2).

Interestingly, mRNAs with an elevated Δ RD compared to the bulk of mRNAs in WT cells only (red, Fig. 2A–2D) were strongly enriched for the category ‘p53 signaling pathway’ by KEGG pathway analysis (Fig. 2E), and indeed the majority of these mRNAs (14/21) belong to the list of 116 high confidence p53 target genes assembled by Fischer [32], including CDKN1A (p21-CIP1), MDM2, BBC3, BTG2, SESN1, SESN2 and FAS (Supplementary Table S1). Hence, it appears that the mRNAs of many transcriptional p53 target genes also benefit from a p53-dependent advantage in translation.

The 5 mRNAs with an elevated Δ RD in both WT and TP53^−/−^ cells (yellow, Fig. 2A–2D) indicate that there is also a small group of mRNAs whose translation is elevated during the DNA damage response in a p53-independent manner. These mRNAs (BIRC3, CXCL8, ATF3, TNFAIP3 and RELB) appear to be mostly related to the inflammatory response. Moreover, our analysis revealed mRNAs with an elevated Δ RD in TP53^−/−^ cells only (green, Fig. 2A–2D), most likely representing mRNAs whose translation is normally attenuated by a p53-dependent translational suppressor mechanism. KEGG pathway analysis showed that these mRNAs are enriched for the categories ‘NF-kappa B signaling pathway’ and ‘NOD-like receptor signaling pathway’ (Fig. 2E), suggesting that these pathways are negatively regulated by p53, or activated by the absence of p53. In fact, an extensive cross-talk between p53 and NF-kappa B signalling has been observed in many studies (reviewed in [33]).

Lastly, our analysis also revealed mRNAs whose translation appears to be suppressed during the DNA damage response (Supplementary Table S1). For at least one of them, NAIP, the mechanism is clearly p53-independent (Fig. 2A–2D).

### Preferential translation of MDM2, SESN1 and CDKN1A mRNAs

**Figure 3.**
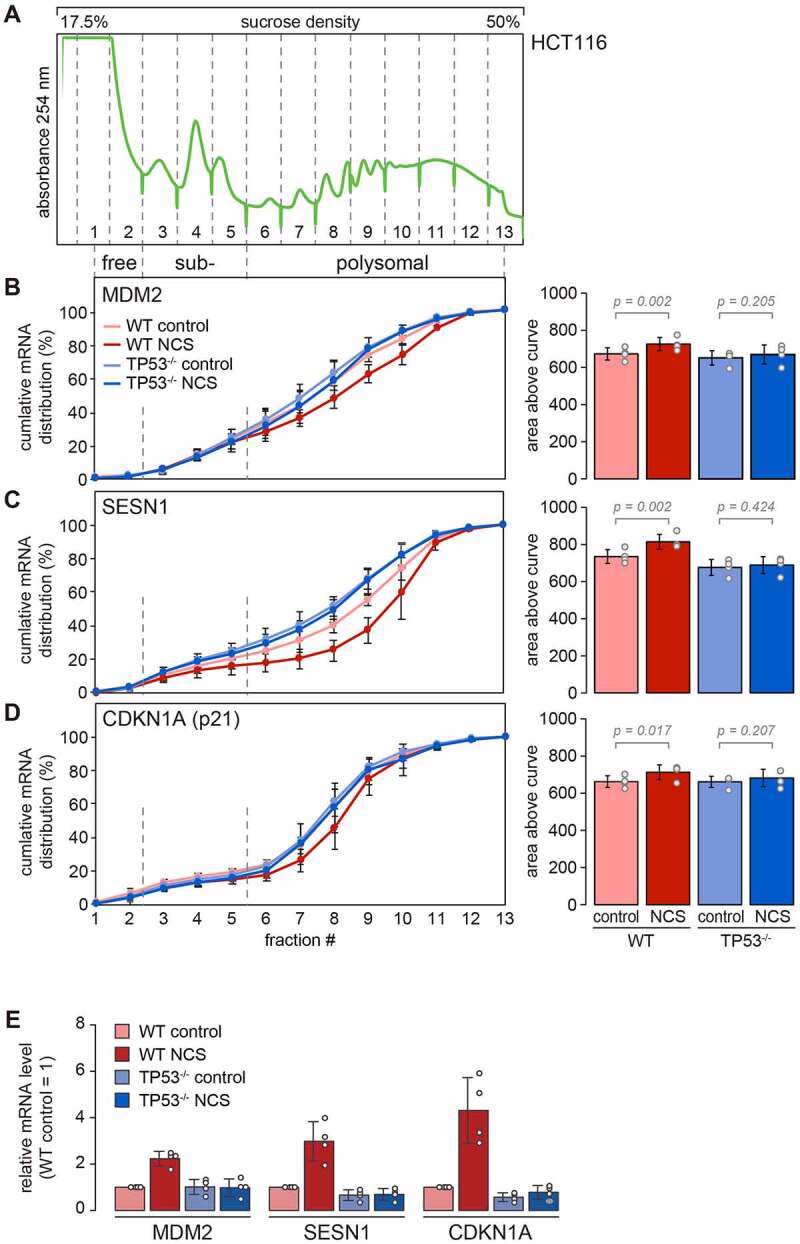
**p53-dependent translational advantage of MDM2, SESN1 and CDKN1A mRNAs**. (A) HCT116-WT and TP53^−/−^ cells were kept under control conditions or treated with NCS (0.2 µg/ml) for 4 h, and polysome profiles were recorded followed by fractionation of the sucrose density gradients. A profile of WT cells under control conditions is represented as an example. (B)–(D) Total RNA was recovered from the polysome fractions, and the distribution of (B) MDM2, (C) SESN1 and (D) CDKN1A mRNA across the polysome gradients was analysed by RT-qPCR. The percentage of mRNA in each fraction is depicted as a cumulative distribution (mean ± SD, n = 4). For each condition, the area above the curve was calculated as a measure for the overall association of the mRNA with ribosomes, and depicted on the right side (mean ± SD). P-values were calculated by one-way ANOVA. (E) Relative mRNA levels of MDM2, SESN1 and CDKN1A were measured by RT-qPCR under the four conditions; the level in WT control cells was set to 1 (mean ± SD, n = 4).

For the remainder of our study, we focused on three transcriptional target genes of p53 for which our Ribo-Seq analysis indicated preferential translation: MDM2, SESN1 and CDKN1A. To confirm that these mRNAs are translated more efficiently upon p53 activation, we measured their distribution across polysome profiles by RT-qPCR in control and NCS treated cells (Fig. 3A). Indeed, all three mRNAs shifted towards heavier polysome fractions upon NCS treatment in WT cells, but not in TP53^−/−^ cells (Fig. 3B–3D). The effect was most pronounced for SESN1 mRNA, where the absence of p53 led to reduced polysome association even under basal control conditions. As expected, the levels of all three mRNAs were elevated upon NCS treatment in a strictly p53-dependent manner (Fig. 3E).

To validate these results, we examined polysome profiles of another colorectal cancer cell line, RKO, using both WT cells and the RKO-TP53^−/−^ counterpart [34]. Since RKO cells respond more slowly to NCS treatment (Supplementary Figure S4A), we used 0.4 μg/ml NCS and a 5.5 h time point for this analysis. All three mRNAs, MDM2, SESN1 and CDKN1A, were associated with lighter fractions in the RKO-TP53^−/−^ cells as compared to RKO-WT cells (Supplementary Figures S4B–S4D). Moreover, SESN1 mRNA shifted to heavier polysome fractions upon NCS treatment in RKO-WT but not in RKO-TP53^−/−^ cells (Supplementary Figure S4C). Likewise, we observed a strong shift of RPL28 mRNA to lighter fractions upon NCS treatment in RKO-WT cells, whereas the control mRNA PRKAB1 was not affected by NCS treatment. From these results we concluded that p53-dependent changes in translation observed in HCT116 cells can at least partially be reproduced in RKO cells.

We then tested whether translational changes would also occur with another DNA damage-inducing drug, and examined the response of HCT116 cells to doxorubicin treatment (Supplementary Figure S5A). While MDM2 and CDKN1A mRNA shifted only very weakly to heavier polysome fractions upon doxorubicin treatment in HCT116-WT cells, SESN1 showed a strong, p53-dependent shift towards heavier fractions (Supplementary Figures S5B–S5D). These results confirm that p53 activation provides a translational advantage for some of its direct target genes, an effect that is most prominently observed for SESN1.

### Analysis of nascent protein synthesis upon p53 induction

**Figure 4.**
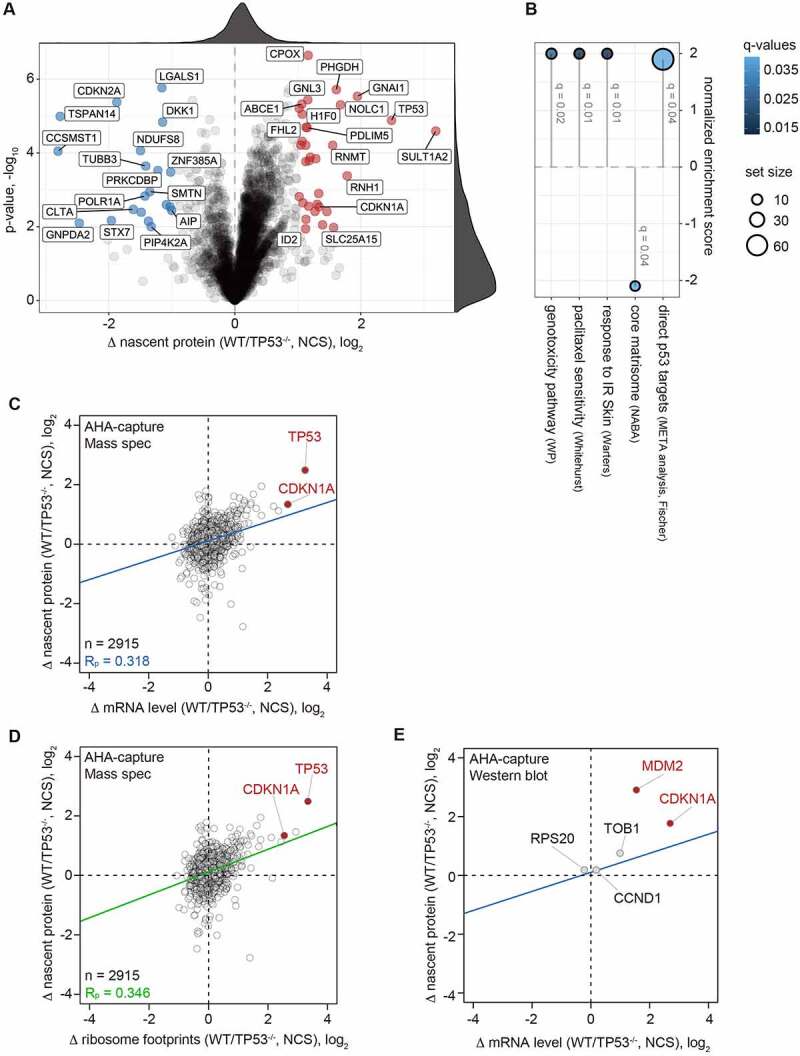
**Nascent proteomics analysis in NCS treated HCT116-WT and TP53^−/−^ cells**. (A) HCT116-WT and TP53^−/−^ cells were treated for 2 h with NCS (0.2 µg/ml), and subjected to both AHA and pSILAC labelling in presence of NCS for additional 4 h. The volcano plot depicts log_2_-transformed fold differences of newly synthesized proteins between WT and TP53^−/−^ cells, covering 3,083 proteins with a minimum of 2 normalized SILAC ratios in the 3 replicates. Proteins with a log_2_ fold difference > 1 and adj. p-value < 0.05, representing proteins with a higher synthesis rate in WT cells, are highlighted in red. Proteins with a log_2_ fold difference < −1 and adj. p-value < 0.05, representing proteins with a higher synthesis rate in TP53^−/−^ cells, are highlighted in blue. (B) Gene set enrichment analysis on newly synthesized proteins in NCS-treated HCT116-WT versus TP53^−/−^ cells. A positive normalized enrichment score indicates elevated synthesis in HCT116-WT cells. Significantly enriched gene sets (q-value < 0.05) from the Molecular Signatures Database are shown together with the number of identified proteins in the respective gene set (set size) and the q-value. (C) Scatter plot of the log_2_ fold difference in nascent protein synthesis between NCS-treated HCT116-WT and TP53^−/−^ cells in relation to the log_2_ fold difference in mRNA expression levels determined by RNA-Seq between HCT116-WT and TP53^−/−^ cells after 4 h of NCS treatment. (D) Scatter plot of the log_2_ fold difference in nascent protein synthesis between NCS-treated HCT116-WT and TP53^−/−^ cells in relation to the log_2_ fold difference in ribosome footprints determined by Ribo-Seq between HCT116-WT and TP53^−/−^ cells after 4 h of NCS treatment. For (C) and (D), genes with fewer than 5 reads (normalized to library size) in the RNA-Seq analysis of NCS-treated HCT116-WT cells were excluded from the analysis. (E) HCT116-WT and TP53^−/−^ cells were treated for 2 h with NCS (0.2 µg/ml) and subjected to AHA labelling in presence of NCS for additional 4 h. Following AHA capture, nascent proteins were quantified by Western blot analysis (n = 3), and the log_2_ fold difference in nascent protein synthesis between NCS-treated HCT116-WT and TP53^−/−^ cells was plotted in relation to the log_2_ fold difference in mRNA expression levels determined by RNA-Seq between HCT116-WT and TP53^−/−^ cells after 4 h of NCS treatment. The blue line represents the regression from panel (C).

To further explore p53-dependent changes in newly synthesized proteins, we made use of a metabolic labelling approach based on a previously published workflow [35], which combines pulsed stable isotope-labelling (pSILAC) and L-azidohomoalanine (AHA)-based labelling of newly synthesized proteins with subsequent enrichment of labelled proteins by click-chemistry. NCS-treated HCT116-WT and TP53^−/−^ cells were simultaneously labelled with AHA and heavy or intermediate SILAC medium. Newly synthesized proteins were then quantified by liquid chromatography followed by tandem mass spectrometry (LC-MS/MS). Nascent protein intensities showed a very high correlation between replicates (Supplementary Figure S6), demonstrating reliable detection of proteins. Thereby, we could quantify 3,083 proteins with a minimum of 2 normalized SILAC ratios in the 3 replicates (Fig. 4A, Supplementary Table S3), indicating multiple proteins that were altered in protein synthesis, including p53 as the positive control.

Gene set enrichment analysis between the NCS-treated HCT116-WT and TP53^−/−^ cells revealed elevated synthesis of proteins encoded by p53 target genes as well as proteins related to genotoxicity, chemotherapy (paclitaxel) resistance and response to ionizing radiation (Fig. 4B), corresponding to reported functions of p53. Besides p53 itself, the synthesis of CDKN1A was also found to be elevated in HCT116-WT cells compared to the TP53^−/−^ counterparts (Fig. 4A). Nascent MDM2 and SESN1 proteins were not detected in our proteomics analysis, presumably because their expression levels are too low.

We then compared the difference in nascent protein production to the difference in mRNA levels between WT and TP53^−/−^ cells, and observed an overall correlation coefficient (R_P_) of 0.318 (Fig. 4C). When nascent protein production was compared to the difference in ribosome footprints, R_P_ was slightly higher at 0.346 (Fig. 4D). Notably, CDKN1A was above the regression line in both analyses, indicating that CDKN1A benefits from a translational advantage in the presence of p53.

We then made use of AHA labelling and capture to determine nascent protein production by Western blot analysis. Given the translation-dependent incorporation of AHA (Supplementary Figure S7A), nascent proteins could be detected specifically by this protocol. As above, we chose an AHA labelling period from 2 to 6 hours after NCS treatment. While there was no suitable antibody for SESN1, we observed a strong increase in MDM2 and CDKN1A (p21-CIP1) synthesis in HCT116-WT but not TP53^−/−^ cells (Supplementary Figure S7B). When the difference in nascent protein synthesis between NCS-treated HCT116-WT and TP53^−/−^ cells was plotted against the difference in mRNA expression (Fig. 4E), the difference in MDM2 and CDKN1A synthesis was clearly above the regression line defined in the nascent proteomics experiment (Fig. 4C). We also included three proteins (TOB1, RPS20 and CCND1 (CyclinD1)) for which our Ribo-Seq analysis did not provide evidence for a p53-dependent translational advantage (Supplementary Table S2), and indeed their position was very close to the regression line (Fig. 4E). Hence, our nascent proteomics analysis is consistent with the result of our Ribo-Seq (Fig. 2) and polysome profile analysis (Fig. 3).

### Isoform-specific translational activation of SESN1 by p53

Since p53 target genes frequently harbour multiple transcription start sites (TSSs) [36] and thereby generate distinct transcript isoforms, we wondered whether translational activation by p53 was isoform-specific. In fact, isoform-specific regulation of translation is well documented for MDM2, which has two validated TSSs generating two transcript isoforms with distinct 5ʹUTRs and different translation efficiencies [20,21].

**Figure 5.**
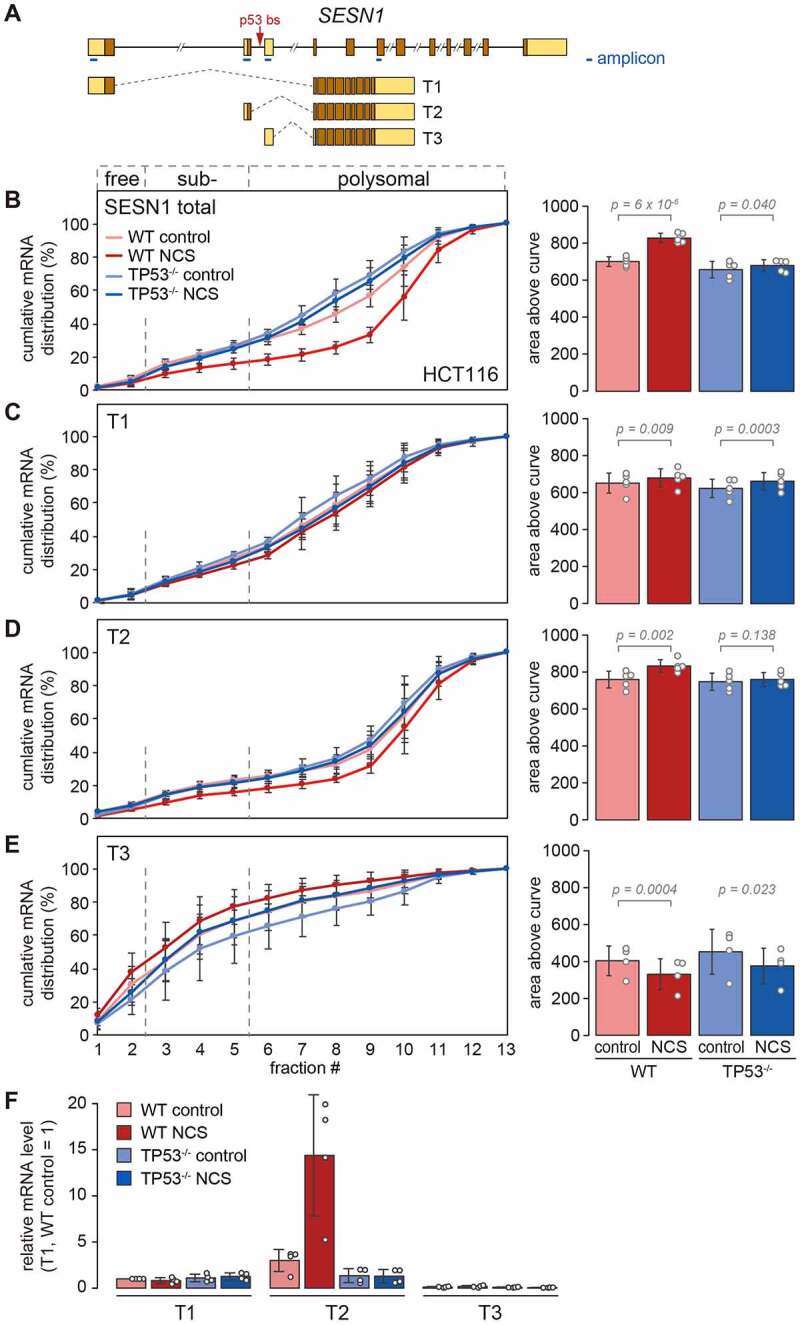
**Isoform-specific translational advantage of T2-SESN1**. (A) Schematic diagram of the *SESN1* gene and its transcript isoforms T1, T2 and T3. Introns are depicted as lines, exons as boxes with untranslated regions (UTRs) in beige and coding regions in brown. The p53 binding site (bs) position is indicated by a red arrow, and the position and length of each amplicon is indicated as blue bar. (B)–(E) HCT116-WT and TP53^−/−^ cells were kept under control conditions or treated with NCS (0.2 µg/ml) for 4 h, and polysome profiles were recorded followed by fractionation of the sucrose density gradients. Total RNA was recovered from the polysome fractions, and the distribution of (B) total SESN1, (C) T1-SESN1, (D) T2-SESN1 and (E) T3-SESN1 mRNA across the polysome gradients was analysed by RT-qPCR. The percentage of mRNA in each fraction is depicted as a cumulative distribution (mean ± SD, n = 4 or 5). For each condition, the area above the curve was calculated as a measure for the overall association of the mRNA with ribosomes, and depicted on the right side (mean ± SD). P-values were calculated by one-way ANOVA. (F) Relative mRNA levels of T1-SESN1, T2-SESN1 and T3-SESN1 were measured by RT-qPCR under the four conditions; the level of T1-SESN1 in WT control cells was set to 1 (mean ± SD, n = 4).

SESN1 has 3 TTSs, thereby expressing T1- (NM_014454.3), T2- (NM_001199933.2) and T3-SESN1 mRNA (NM_001199934.2), all of which contain different translation start sites and unique 5ʹUTR sequences (Fig. 5A). p53 binds to the second intron and induces transcription of T2- and T3-SESN1 [37]. To test if SESN1 isoforms might have different translation efficiencies, we designed primers specific for each SESN1 isoform (Fig. 5A, amplicons indicated in blue) and analysed the distribution of the corresponding mRNAs across polysome profiles by RT-qPCR (Fig. 5B–5E). As seen above (Fig. 3C), total SESN1 mRNA shifted strongly towards heavier fractions in WT but not TP53^−/−^ cells (Fig. 5B). In contrast, the T1 isoform showed only a slight shift towards heavier fractions with no difference between WT and TP53^−/−^ cells (Fig. 5C). Interestingly, T2-SESN1 mRNA is enriched in heavier polysome fractions under control conditions as compared to total SESN1 mRNA, and shifted further into the heavy fractions upon NCS treatment in a p53-dependent manner (Fig. 5D). Hence, T2-SESN1 appears to be the translationally more active isoform. The third isoform, T3-SESN1 mRNA, was mainly recovered from sub-polysomal fractions and shifted towards lighter fractions upon NCS treatment in both WT and TP53^−/−^ cells (Fig. 5E). Thus, T3-SESN1 seems to be poorly translated and further repressed upon NCS treatment independently of p53.

We then estimated the relative expression level of the three isoforms based on RT-qPCR from total RNA. While T1-SESN1 was expressed constitutively, T2- and T3-SESN1 were induced in a p53-dependent manner by NCS treatment, though the expression level of T3-SESN1 remained extremely low (Fig. 5F). Taken together, our results demonstrate that translational activation of SESN1 relies on the induction of the T2-SESN1 isoform, which is translated more actively than the two other isoforms. Moreover, the shift in polysome prolife distribution revealed that there is an additional *trans*-acting and p53-dependent mechanism that further enhances translation of the T2-SESN1 isoform during the DNA damage response.

### Translational activation of multiple CDKN1A isoforms by p53

**Figure 6.**
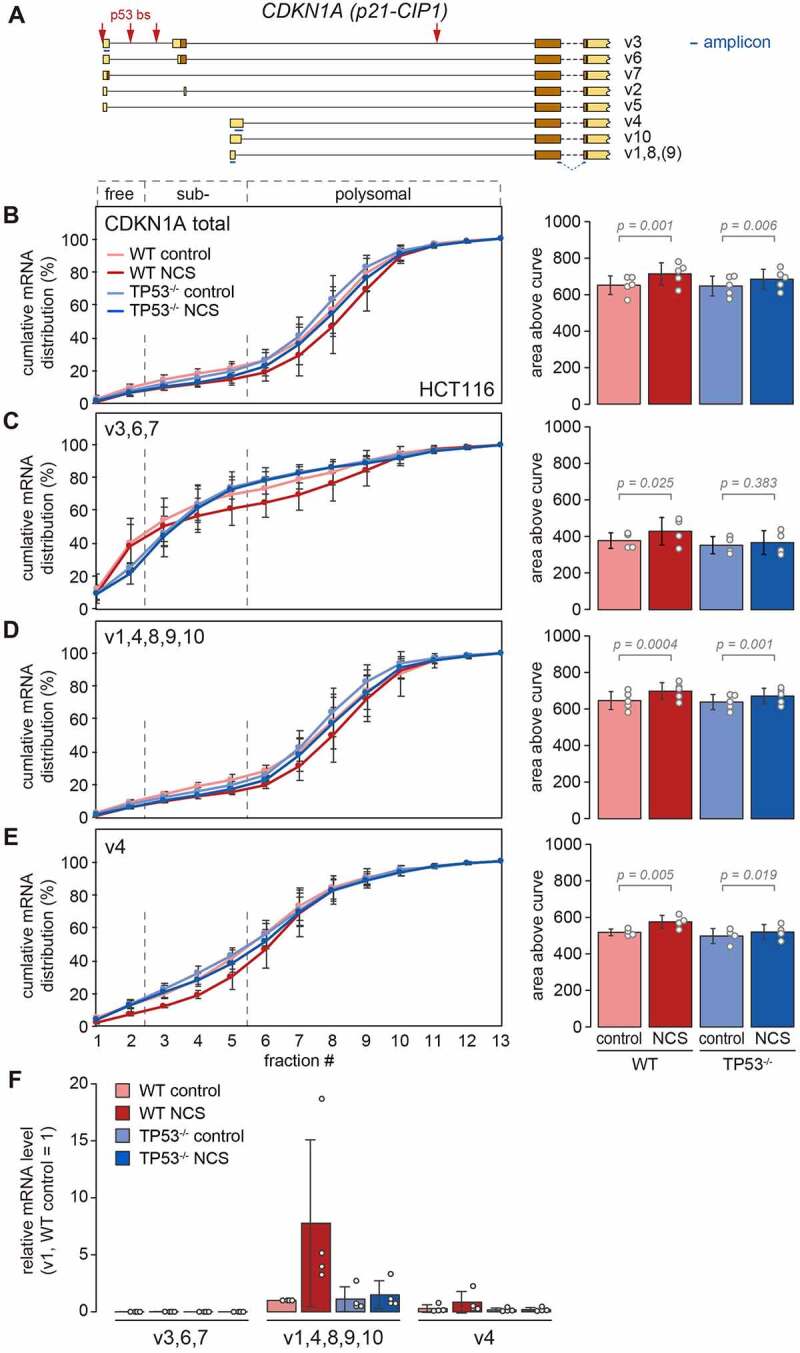
**Translational advantage of multiple CDKN1A isoforms**. (A) Schematic diagram of the first four exons of *CDKN1A* transcript isoforms v1, v2, v3, v4, v5, v6, v7, v8, v9 and v10. Introns are depicted as lines, exons as boxes with UTRs in beige and coding regions in brown. p53 bs positions are indicated by red arrows, and the position and length of each amplicon is indicated as blue bar. (B)–(E) HCT116-WT and TP53^−/−^ cells were kept under control conditions or treated with NCS (0.2 µg/ml) for 4 h, and polysome profiles were recorded followed by fractionation of the sucrose density gradients. Total RNA was recovered from the polysome fractions, and the distribution of (B) total CDKN1A mRNA, (C) CDKN1A isoforms [v3,6,7], (D) CDKN1A isoforms [v1,4,8,9,10] and (E) CDKN1A isoform [v4] mRNA across the polysome gradients was analysed by RT-qPCR. The percentage of mRNA in each fraction is depicted as a cumulative distribution (mean ± SD, n = 4 or 5). For each condition, the area above the curve was calculated as a measure for the overall association of the mRNA with ribosomes, and depicted on the right side (mean ± SD). P-values were calculated by one-way ANOVA. (F) Relative mRNA levels of CDKN1A isoforms [v3,6,7], CDKN1A isoforms [v1,4,8,9,10] and CDKN1A isoform [v4] were measured by RT-qPCR under the four conditions; the level of isoforms [v1,4,8,9,10] in WT control cells was set to 1 (mean ± SD, n = 4).

According to NCBI GenBank, CDKN1A has two annotated TSSs and several splicing isoforms that give rise to eight different 5ʹUTR variants (Fig. 6A). We designed primer sets to detect two groups of isoforms, [v3,6,7] and [v1,4,8,9,10], as well as a primer pair specific for v4-CDKN1A (Fig. 6A, amplicons indicated in blue). We then examined the distribution of these isoforms across polysome profiles by RT-qPCR (Fig. 6B–6E). As in Fig. 3D, total CDKN1A mRNA showed a small though consistent shift towards heavier fractions upon NCS treatment (Fig. 6B). Although this was statistically significant in both HCT116-WT and TP53^−/−^ cells, the shift was stronger in WT cells (Fig. 6B), suggesting the existence of a p53-dependent and a p53-independent mechanism. The [v3,6,7] isoforms were mostly recovered from sub-polysomal fractions (Fig. 6C), showing that transcripts originating from the upstream promoter are poorly translated. Nonetheless, the [v3,6,7] isoforms showed a p53-dependent shift towards heavier fractions upon NCS treatment (Fig. 6C), indicative of translational activation.

The CDKN1A mRNA isoforms transcribed from the downstream TTS, [v1,4,8,9,10], had a polysome distribution similar to that of total CDKN1A mRNA, including a slight shift towards heavier fractions upon NCS treatment (Fig. 6D). Among these isoforms, we could separately detect v4-CDKN1A mRNA, which has a longer 5ʹUTR containing a uORF that was previously described to repress translation [38]. The v4-CDKN1A mRNA was mostly enriched in sub-polysomal and light polysome fractions, and again showed a shift towards heavier fraction upon NCS treatment in a p53-dependent manner (Fig. 6E). The analysis of relative expression levels showed that p53 activation led to a prominent transcriptional induction of the isoforms from the downstream TTS, [v1,4,8,9,10], and that these isoforms were generally expressed at higher levels than the isoforms from the upstream TSS, [v3,6,7] (Fig. 6F). Taken together, our results demonstrate that multiple CDKN1A mRNA isoforms transcribed from both the up- and the downstream promoter are translationally activated in a p53-dependent, and, to a lesser degree, also p53-independent manner, both representing *trans*-acting mechanisms that ensure preferential translation of p21-CIP1.

## Discussion

Several studies have previously addressed the impact of p53 on translation of individual mRNAs using genome-wide approaches, either based on polysome profiling [22–24] or Ribo-Seq [15]. These analyses typically used long-term activation of p53, e.g. by treatment with Nutlin-3a for 12–16 h, and identified a large number of mRNAs whose association with polysomes changed disproportionately compared to the change in mRNA levels. Our study focused on short-term activation of p53 using 4 h treatment with NCS, a condition at which gene expression is far from steady state due to the acute DNA damage response including the transcriptional impact of p53. By setting changes in RD in relation to changes in mRNA levels, we were able to separate mRNAs subject to active translational control from those whose RD changes due to the kinetic (passive) effect that results from a change in the level of the mRNA (Fig. 2A and 2B). This allowed us to delineate 26 mRNAs whose RD was elevated in relation to the kinetic effect (distance from the regression line > 1), indicative of translational activation (Fig. 2A–2D, Supplementary Table S1). For 21 of these mRNAs, the translational effect was dependent on p53, and the majority of them (14) are transcribed from bona-fide p53 target genes. This indicates that translational up-regulation early after p53 activation serves to amplify the transcriptional effect of p53, and make sure that the newly synthesized mRNAs are preferentially translated.

Different from previous studies that pointed to hundreds of mRNAs being translationally regulated by p53 [15,22–24], our analysis of ribosomal footprints provides evidence for much smaller groups of 21 and 18 mRNAs that have a translational advantage and disadvantage, respectively, in a p53-dependent manner (Supplementary Table S1). This may be due to the fact that we focused on an early time point (4 h) after p53 activation, which reduces secondary effects, and that our analysis separates passive from active changes in RD.

One mechanism by which p53 influences the translation of mRNAs is through the induction of alternative isoforms that differ in their translation efficiency from constitutively expressed isoforms. A paradigm for this mechanism was set by early research on MDM2, which harbours two TSSs originating from a P1 and a P2 promoter. P1 is the constitutive promoter, whereas p53 activates transcription from the downstream P2 promoter, generating two transcript isoforms with distinct 5ʹUTRs. While the 5ʹUTR of P1-MDM2 contains two upstream open reading frames (uORFs) that reduce the efficiency of translation initiation, the 5ʹUTR of P2-MDM2 lacks uORFs and is translationally more active [20,21]. Hence, p53-induced expression of the translationally more active P2-MDM2 isoform is an elegant mechanism to couple transcriptional induction with efficient synthesis of the corresponding protein.

Here, we describe SESN1 as a second case where p53 induces expression of a translationally advantageous isoform (Fig. 5). While the constitutive isoform T1-SESN1 is associated with light polysomes, the p53-induced isoform T2-SESN1 is associated with heavier polysomes, indicative of a higher RD and more efficient translation. A third isoform, T3-SESN1, was barely expressed at mRNA level, and very poorly translated according to the polysome distribution. Indeed, both T1- and T3-SESN1 have putative uORFs in their 5ʹUTRs, which may explain their reduced translation efficiency. Notably, the polysome profile analysis of T2-SESN1 revealed not only that this isoform is translated more efficiently than T1- and T3-SESN1, but also that p53 activation further enhances the translation of T2-SESN1 (Fig. 5D). Thus, we postulate that an additional, *trans*-acting mechanism augments the synthesis of T2-SESN1. Interestingly, the T1- and T2-SESN1 isoforms generate proteins with different N-termini, and the T2-SESN1 protein (Sesn1S) appears to associate more efficiently with the GATOR2 complex than T1-SESN1 (Sesn1L) [10]. This indicates that SESN1 is regulated at multiple layers including a sophisticated transcriptional switch by which a more efficiently translated mRNA is induced, encoding a more effective protein isoform whose synthesis is further enhanced by a *trans*-acting mechanism.

For CDKN1A, we also observed a combination of p53-induced transcription of translationally more active isoforms, and a *trans*-acting mechanism that appears to enhance the translation efficiency of all isoforms (Fig. 6). The [v3,6,7] isoforms originating from the upstream promoter were expressed very poorly at the mRNA level, and their association with polysomes was weak in general. Nonetheless, p53 activation led to a shift of the [v3,6,7] isoforms into heavier polysomes, indicating translational enhancement by a *trans*-acting mechanism. The [v1,4,8,9,10] isoforms originating from the downstream promoter were expressed at higher levels, prominently induced by p53 activation, and associated with polysomes much more strongly. Again, p53 activation caused a slight shift of the [v1,4,8,9,10] isoforms towards heavier polysomes, suggesting that these isoforms benefit from the same *trans*-acting mechanism enhancing CDKN1A mRNA translation.

Interestingly, translational activation of CDKN1A was also observed upon UV-irradiation of keratinocytes [39]. In this case, the 5ʹUTR of the v4 isoform was found to mediate translational activation, through a mechanism that depends on eIF2α phosphorylation but is independent of two uORFs present in the v4 5ʹUTR [39]. In our system, treatment of HCT116 cells with NCS led to translational activation of all CDKN1A isoforms and was not specific for v4. Also, NCS treatment did not lead to any notable change in eIF2α phosphorylation (Fig. 1D), indicating that the *trans*-acting mechanism we observe is different from the one described upon UV-irradiation. Given that the CDKN1A isoforms we analysed differ in their 5ʹUTRs, it is reasonable to assume that the *trans*-acting mechanism of translational activation affecting all isoforms involves the coding region or 3ʹUTR of CDKN1A.

An interesting question to speculate about is the nature of the *trans*-acting mechanism by which p53 enhances the translation of several of its target mRNAs. A first possibility is that p53 may directly bind to specific mRNAs and control their translation. In fact, there is evidence that p53 binds to the 5ʹUTR of FGF2 mRNA [18,19, 40], MDMX mRNA [17] and its own mRNA [16], though in each case this was associated with translational suppression of the bound mRNA. Also, it should be noted that concerns have been raised as to whether p53 can indeed interact with RNA in a sequence-specific manner, and whether RNA-binding is relevant for the function of p53 in cells [26,41].

A second possibility is that p53 may regulate the translation of specific mRNAs by controlling the expression of miRNAs, which in turn promote the degradation and/or repress the translation of target mRNAs [42]. Indeed, p53 was shown to induce the expression of numerous miRNAs [43]. Since miRNAs typically act as repressors of mRNA expression, this mechanism primarily accounts for the inhibition of mRNA stability and/or translation upon p53 activation [43], but is unlikely to explain preferential translation upon p53 activation.

A third possibility is that p53 regulates mRNAs post-transcriptionally through induction of certain RNA-binding proteins (BPs), which in turn affect the translation of mRNAs or other aspects of their life cycle. Indeed, there are a few RNA-BPs among high confidence p53 target genes [32] including the mRNA decapping enhancer DCP1B, the mRNA editing enzyme APOBEC3C and the ribosomal protein RPS27L. Recently, Rizotto *et al*. identified a GC-rich motif in the 3ʹUTR of several mRNAs whose translation is enhanced upon stabilization of p53 by Nutlin-3a in SJSA1 but not in HCT116 cells [25]. Moreover, the authors could show that the RNA helicase DHX30 and the RNA-BP PCBP2 are involved in suppressing the translation of these mRNAs in HCT116 cells [25]. Given that neither DHX30 nor PCBP2 appear to be regulated by p53 activation, they probably do not account for the *trans*-acting mechanism by which p53 target genes such as SESN1 and CDKN1A are preferentially translated. Nonetheless, we anticipate that future work will unravel RNA-BPs responsible for enhancing the translation of specific mRNAs in response to p53 activation.

## Materials and methods

### Cell culture and reagents

The HCT116-TP53^−/−^ [30] and RKO-TP53^−/−^ cell lines [34] were generated in the lab of Bert Vogelstein (Johns Hopkins School of Medicine, Baltimore, Maryland, USA). HCT116 and RKO cells were maintained in Dulbecco’s Modified Eagle Medium (DMEM, Gibco) containing 10% foetal bovine serum (Sigma-Aldrich), 2 mM L-glutamine, 100 U/ml penicillin and 0.1 mg/ml streptomycin (all PAN Biotech) at 37°C in 5% CO_2_. Cells were seeded 1 or 2 days before the experiment in 10-cm dishes and grown to 60–80% confluency. Cells were then treated with neocarzinostatin (NCS, Sigma-Aldrich) at a concentration of 0.2 µg/ml for 4 h (HCT116 cells) or 0.4 µg/ml for 5.5 h (RKO cells). For control, cells were treated with an equal volume of phosphate buffered saline (PBS). During puromycin incorporation assays, cells were treated with puromycin (Gibco) at a concentration of 1 µg/ml, or H_2_O alone as control, for 5 min prior to cell lysis.

### Antibodies

The following antibodies were used for Western blot analysis and immunofluorescence microscopy: monoclonal mouse anti-p53 (DO-1, Santa Cruz, sc-126), polyclonal rabbit anti-p21-CIP1 (Santa Cruz, sc-397), monoclonal mouse anti-phospho-Ser139-H2AX (γH2AX) (JBW301, Millipore, 05–636), monoclonal mouse anti-MDM2 (Abcam, ab16895), polyclonal rabbit anti-phospho-Ser51-eIF2α (Cell Signaling, #9721), polyclonal rabbit anti-eIF2α (Cell Signaling, #9722), monoclonal mouse anti-puromycin (Millipore, MABE343), polyclonal rabbit anti-β-actin (Abcam, ab8227-50), polyclonal rabbit anti-TOB1 (Proteintech, 14915-1-AP), monoclonal rabbit anti-RPS20 (Abcam, ab133776) and monoclonal rabbit anti-Cyclin D1 (EPR2241, Abcam, ab134175).

### Polysome profiling

Cells grown in 10-cm dishes were washed once with ice-cold PBS containing 100 µg/ml cycloheximide (CHX) and directly lysed using 290 µl of polysome lysis buffer (20 mM Tris-HCl, pH 7.5, 150 mM NaCl, 5 mM MgCl_2_, 1 mM DTT, 100 µg/ml CHX, 1% Triton X-100, 40 U/ml RNasin (Promega) supplemented with EDTA-free complete protease inhibitors (Roche)), scraped and harvested into microtubes. Lysates were rotated end-over-end at 4°C for 10 min and centrifuged at 10,000 × *g* for 10 min at 4°C to remove nuclei and cell debris. The concentration of lysates was adjusted by measuring the absorbance at 260 nm, and equal amounts (250 µl) were loaded on top of 3.95 ml 17.5–50% sucrose density gradients, for which sucrose was dissolved in a buffer containing 20 mM Tris-HCl, pH 7.5, 5 mM MgCl_2_, and 150 mM NaCl. Gradients were subjected to ultracentrifugation at 40,000 rpm using a SW60 rotor (Beckman) for 105 min at 4°C. Fractions were eluted from the top of the gradient and polysome profiles were recorded by continuously measuring the absorbance at 254 nm using a Teledyne ISCO Foxy RI system in combination with PeakTrak software. To quantify the percentage of monosomal and polysomal ribosomes, the area under the curve corresponding to monosomal, polysomal and total ribosomes was integrated.

### Ribo-Seq and RNA-Seq analysis

Cells grown in 10-cm dishes were washed once with ice-cold PBS containing 100 µg/ml CHX and directly lysed using 290 µl of Ribo-Seq lysis buffer (20 mM Tris-HCl, pH 7.5, 10 mM MgCl_2_, 200 mM KCl, 2 mM DTT, 100 µg/ml CHX, 1% NP-40, 40 U/ml RNasin (Promega), EDTA-free complete protease inhibitors (Roche)). The total RNA concentrations between samples were adjusted by measuring absorbance at 260 nm. From the adjusted cellular lysates, 50 µl was taken and diluted with 250 µl of Ribo-Seq lysis buffer for preparing input samples to be analysed by RNA-Seq. The remaining lysate (approximate volume of 250 µl) was treated with 240 U/1 A260 of RNase I (Ambion) for 5 min at 4°C on a rotator, and then loaded on top of 3.95 ml 17.5–50% sucrose density gradients for which sucrose was dissolved in a buffer containing 20 mM Tris-HCl, pH 7.5, 5 mM MgCl_2_, and 150 mM NaCl. Centrifugation and profile recording were carried out as indicated in the above section, and fractions of 300 µl each were collected with the fraction collector that is integrated in the Teledyne ISCO Foxy RI system. Fractions corresponding to monosomes and disomes were used for preparation of ribosome protected fragments (RPFs).

RNA was then purified from the input and RFP samples by first mixing with an equal volume (300 µl) of urea buffer (10 mM Tris-pH 7.5, 350 mM NaCl, 10 mM EDTA, 1% SDS, 7% urea) and incubation with 300 µl phenol:chloroform:isamylalcohol (25:24:1) for 10 min at 65°C. The RNA was extracted upon centrifugation for 10 min at 14,000 × *g* by recovery of the aqueous phase followed by precipitation using 600 µl isopropanol and 1 µl of GlycoBlue (Invitrogen) at −20°C overnight. After washing with 70% ethanol, the RNA was dissolved in 10 µl H_2_O.

Prior to library preparation, ribosomal RNA was depleted from all samples using the Ribo-Zero Gold Kit (Illumina). Input RNA was randomly fragmented by alkaline hydrolysis at pH 10.0 for 12 min at 95°C. Fragmented RNA and RPFs were then size-selected (25–35 nt) on a 15% polyacrylamide Tris-borate-EDTA-urea gel. The gel pieces were crushed, and RNA was extracted by incubating overnight at 4°C in 300 µl extraction buffer containing 0.3 M NaCl and 80 U RNase OUT (invitrogen). Gel pieces were removed by centrifugation at 14,000 × *g* for 5 min at 4°C through a 0.45 µm pore size membrane (Nanosep MF tubes, Pall), and RNA was precipitated by addition of 1 µl GlycoBlue (Invitrogen) and 300 µl isopropanol. After washing with 70% ethanol, the RNA was dissolved in 14 µl H_2_O and subjected to end-repair with T4 polynucleotide kinase (NEB) for 1 h at 37°C. Following an additional round of purification and precipitation using isopropanol, 1–20 ng RNA were used for library preparation using the NEXTflex Small RNA-Seq Kit v3 (PerkinElmer) according to the manufacturer’s manual. Libraries were multiplexed and sequenced with a NextSeq 500 sequencer (Illumina).

### Analysis of Ribo-Seq and RNA-Seq data

Adapters were removed with the FASTX-toolkit (http://hannonlab.cshl.edu/ fastx_toolkit/), retaining only sequences that are at least 28 nt long. The four random nucleotides at the beginning and the end of the reads were trimmed with an in-house developed perl script. Alignment was performed with bowtie v1.2.2 [44] allowing a maximum of two mismatches and reporting all alignments in the best stratum (settings: -a – best – stratum – v 2). Reads that did not map to tRNA or rRNA sequences (as downloaded from the UCSC Genome Browser) were aligned to a human transcriptome (Gencode V27 as downloaded from the UCSC Genome Browser wgEncodeGencodeBasicV27 table). Reads were filtered with in-house developed perl scripts to retain only reads that are between 25 and 35 nt long and map to ORFs of isoforms arising from one specific gene (as defined by a common gene symbol). An offset of 12 nt upstream of the start codon and 15 nt upstream of the stop codon with respect to the 5’ end of the read was assumed. Normalization was performed using size factors obtained with the median ratio method of the DESeq2 package, v1.18.1 [45]. All scripts used for processing, alignment and analysis of the Ribo-Seq data are available on our OSF project page (https://osf.io/tgrbv/?view_only=5cc5a1725b3c4407a1a9f3348a9da0dc). Genes with less than 10 read counts in the NCS-treated condition in WT or TP53^−/−^ cells were filtered out. For regression analysis, ordinary least squares regression was performed on the mean of the four replicates using the lm() function of R. The genes with an absolute distance > 1 from the regression line in WT or TP53^−/−^ cells in the mean of four replicates were considered as candidates for active translational regulation, coloured in Fig. 2A–2D and listed in Supplementary Table S1. Pathway and gene ontology analyses were performed with Metascape (http://metascape.org) [46].

### Polysome fractionation and quantitative RT-PCR

Cellular lysates were prepared and fractionated by sucrose density gradient centrifugation as described above, without RNase I digestion. Prior to extraction of the RNA from the 13 fractions, 12.5 fmol of an *in vitro* transcribed RNA (mouse β-globin or Renilla luciferase) was added to each fraction. After RNA purification, an equal volume of RNA from each fraction (in the range of 1–2 µg) was used for the RT reaction with M-MLV reverse transcriptase (Promega) and random hexamer primers for 1 h at 37°C. Quantitative PCR (qPCR) was performed using the PowerUp SYBR Green Master Mix (Applied Biosystems). Primers used in these experiments are listed in Supplementary Table S4. To compare the expression levels of each isoform, we normalized them with the following equation:

Isoform (Amplification factor^Ct value)/spike-in control (Amplification factor^Ct value)

### Immunofluorescence microscopy

Cells were seeded on coverslips 1 or 2 days before the experiment and grown to 60–80% confluency. Cells were then washed with PBS, fixed with 4% paraformaldehyde for 10 min, permeabilized with 0.5% Triton X-100 in PBS for 10 min and blocked with 3% BSA in TBST buffer (50 mM Tris-HCl, pH 7.5, 150 mM NaCl and 0.1% Tween®-20) for 1 h. Coverslips were then incubated overnight at 4°C in 125 µl PBS containing primary antibodies at the following dilution: polyclonal rabbit anti-p21 (1:500) and monoclonal mouse anti-phospho-Ser139-H2AX (γH2AX) (1:1,000). After washing with PBS, the coverslips were incubated with Cy3- or Cy2-conjugated secondary antibodies (Jackson ImmunoResearch) diluted 1:1,000 in TBST for 30 min, and DNA was stained with Hoechst dye (1:10,000; Sigma-Aldrich) for 10 min. Finally, cells were embedded with one drop of mounting medium (Invitrogen) on glass slides. Microscopy was performed on a Nikon Eclipse Ti2-E microscope in combination with a 40× oil objective (NA 1.4). Images were captured with a sCMOS pco.edge 4.2 LT camera (PCO) and processed using Fiji and Adobe Photoshop software.

### Nascent protein analysis by combined pSILAC and AHA labelling

For the quantification of nascent proteins, a metabolic labelling approach was used that combines pSILAC and AHA labelling of newly synthesized proteins, subsequent click-chemistry based enrichment of the labelled proteins, liquid chromatography and LC-MS/MS analysis, following a workflow adapted from [35].

HCT116-WT and TP53^−/−^ cells were treated with 0.2 µg/ml NCS for 75 min and subsequently washed with warm PBS and incubated with DMEM high glucose medium deprived of methionine, arginine and lysine for 45 min. Pulsed SILAC and AHA labelling was carried out with methionine-free DMEM high glucose medium containing heavy (^13^C_6_-^15^N_4_-Arg, ^13^C_6_-^15^N_2_-Lys) and intermediate (^13^C_6_-Arg, D_4_-Lys) arginine and lysine, 0.2 µg/ml NCS and 100 µM AHA for 4 h. HCT116-WT cells were labelled with heavy and TP53^−/−^ cells with intermediate SILAC medium. After 4 h labelling, which corresponds to 6 h NCS treatment, cells were washed with cold PBS and cell pellets were shock frozen with liquid nitrogen.

### AHA capture for nascent protein mass spectrometry

Cell pellets were lysed with lysis buffer containing 1% sodium-dodecylsulfate (SDS), 300 mM HEPES (pH 8.0) and complete EDTA-free protease inhibitor cocktail (Merck). The lysates were sonicated with a probe sonicator (Branson) at 10% power for a total duration of 1 min. Cellular debris was removed from the lysates by centrifugation at 20,000 × *g* for 15 min, and protein concentrations were determined using a BCA assay (Thermo Fisher). A total of 1 mg (500 µg per cell type) was used as input for the enrichment. The combined lysates were alkylated with 14.6 mM iodoacetamide (IAA) for 20 min at room temperature. Subsequently, AHA-containing proteins were coupled to 50 µl propargylamine-coupled epoxy-activated magnetic sepharose beads (Cube Biotech) via the addition of 1.15 mM CuSO_4_, 5.77 mM Tris-hydroxypropyltriazolylmethylamine (THPTA), 11.54 mM aminoguanidine HCl and 11.54 mM sodium ascorbate. The reaction mixture was incubated for 2 h at 40°C. The supernatant was discarded and the beads were washed with 1.8 ml milliQ H_2_O. Proteins bound by the beads were reduced by adding 10 mM Tris(2-carboxylethyl)phosphine (TCEP) and 40 mM 2-chloroacetaminde (CAA), dissolved in 100 mM Tris-HCl buffer (pH 8.0) containing 200 mM NaCl, 0.8 mM ethylendiamintetraacetic acid (EDTA) and 0.8% SDS, followed by incubation at 70°C for 20 min and subsequent incubation at 20°C for 15 min. The beads were then washed with 6 ml 1% SDS dissolved in 100 mM Tris-HCl (pH 8.0), 250 mM NaCl and 1 mM EDTA buffer, followed by 2 ml milliQ H_2_O, 6 ml 6 M Guanidine-HCl in 100 mM Tris-HCl (pH 8.0) and 6 ml 20% acetonitrile in milliQ H_2_O. After the washing steps, the beads were resuspended in 200 µl 100 mM Tris-HCl (pH 8.0), 5% acetonitrile and 2 mM CaCl_2_-containing buffer. Proteins coupled to the beads were digested with 1 µg Trypsin/LysC mix (Promega) for 16 h at 37°C. Peptides were desalted using the SP3 peptide clean-up protocol [47]. Purified peptides were then dissolved in 0.1% formic acid and used for LC-MS/MS-based analysis.

### LC-MS/MS

Quantitative measurements of tryptic peptides of the enriched newly-synthesized proteins was carried out using an EASY-nLC 1200 system (Thermo Fischer Scientific) coupled to an Orbitrap Fusion mass spectrometer (Thermo Fischer Scientific).

The peptides were separated by reverse-phase liquid chromatography using an Acclaim PepMap trap column (Thermo Fisher Scientific, C18, 20 mm × 100 μm, 5 μm C18 particles, 100 Å pore size) and a nanoEase M/Z peptide BEH C18 analytical column (Waters, 250 mm × 75 μm 1/PK, 130 Å, 1.7 μm) with 0.1% formic acid (solvent A) and 80% acetonitrile (solvent B) as mobile phase. The samples were loaded onto the trap column with constant flow of solvent A at a maximum pressure of 800 bar. The analytical column was equilibrated with 2 μl solvent A at a maximum pressure of 600 bar heated to 55°C using a HotSleeve+ column oven (Analytical Sales & Services). The peptides were eluted with a constant flow rate of 300 nl/min. Concentration of solvent B was gradually increased during the elution of the peptides. The gradient started with 3% solvent B for the first 4 min, increased to 8% after 4 min and to 10% after 6 min. After 89 min the percentage of solvent B was raised to 32%, and after 101 min to 50%. From 102 min to 109 min of the gradient, the percentage of solvent B increased to 100%. After 110 min the system was re-equilibrated using 3% solvent B for 10 min. The peptides were ionized and injected using the Nanospray flex ion source (Thermo Fischer Scientific) and a Sharp Singularity nESI emitter (ID = 20 µm, OD = 365 µm, L = 7 cm, α = 7.5°) (Fossiliontech), connected to a SIMPLE LINK UNO-32 (Fossiliontech). A static spray voltage of 2.5 kV was applied to the emitter and the capillary temperature of the ion transfer tube was set to 320°C.

The Orbitrap Fusion mass spectrometer was operated in the data-dependent mode using a full scan range of 375–1500 m/z, Orbitrap resolution of 60,000, automatic gain control target of 250%, RF lens set to 60% and maximum injection time of 50 ms. Monoisotopic peak determination was set to peptide mode, dynamic exclusion was set to a 20s duration with 10 ppm tolerance and isotope exclusion, and an intensity threshold of 5 × 10^5^ was set. MS/MS spectra were acquired in the linear ion trap detector in Rapid mode. The quadrupole isolation window was set to 1.6 m/z and HCD collision energy was set to 33%. The total cycle time was fixed to 3 s. The data type for the MS/MS spectra was set to centroid mode.

### Analysis of proteomic data

Raw files were processed using Maxquant version 2.0.1 and the Andromeda search engine [48]. A human proteome fasta file, retrieved from the SwissProt database (version from February 2021 with 20,934 entries) was used for the analysis of the samples. The enzymatic digestion was set to Trypsin/P and a maximum of two missed cleavages per peptide were allowed. The multiplicity was set to 3, comprising of a light channel, an intermediate channel with Arg6 and Lys4 and a heavy channel with Arg10 and Lys8. Cysteine carbamidomethylation was set as fixed modification, whereas methionine oxidation, N-terminal acetylation, lysine acetylation and deamidation of asparagine and glutamine were set as variable peptide modifications. The Re-quantify, match between runs and dependent peptide search options were enabled with default parameters. Unique and razor peptides were used for quantification and normalized SILAC ratios and iBAQ values were calculated. The minimum ratio count was set to 0 to not exclude identifications in single SILAC channels. The PSM and protein FDR threshold was set to 1%. Additionally, LFQ values for the individual SILAC channels were calculated with default settings.

The evidence.txt and proteinGroups.txt output tables were processed in the R software environment (version 4.0.3). Protein groups with a minimum of 2 normalized SILAC ratios in the 3 replicates were used for the quantitative analysis. Statistical analysis of the proteomic data was carried out using the Limma [49] and DEqMS [50] R/Bioconductor packages. The data was fitted onto a linear model and an empirical Bayes moderated t-test was performed. The number of SILAC ratios per protein group was included as a factor for the variance estimation. P-values were adjusted using the Benjamini-Hochberg approach. Geneset enrichment analysis of the proteomic data was carried out using the clusterProfiler [51] R/Bioconductor package. Gene lists of the Molecular Signatures Database were retrieved and analysed using the msigdbr package of the CRAN software repository (https://cran.r-project.org/web/packages/msigdbr/). Gene sets of the Hallmark (H)-, curated gene set (C2), Ontology gene set (C5), regulatory gene set (C3) and computational gene set subcategories were included in the analysis. All quantified proteins were ordered according to the log_2_ fold change values and used as input for the geneset enrichment analysis [52]. P-values were adjusted using the Benjamini-Hochberg approach, and a q-value cut-off of 0.05 was used for the enrichment analysis.

### AHA labelling and capture of nascent proteins for Western blot analysis

HCT116-WT and TP53^−/−^ cells grown on 15 cm dishes (approx. 2.4 × 10^7^ cells/dish) were left untreated or treated with 0.2 µg/ml NCS; first in DMEM medium for 90 min, then in methionine-free RPMI medium for 30 min, and finally in methionine-free RPMI medium (Gibco) containing 0.8 mM AHA (Sigma-Aldrich) for 4 h. As negative controls, we used 0.8 mM methionine (Sigma-Aldrich) instead of AHA, or added 100 µg/ml cycloheximide in presence of 0.8 mM AHA. After labelling, cells were washed with cold PBS, collected in microcentrifuge tubes, and disrupted in 500 µl lysis buffer (115 mM Tris-HCl, pH 8.5, 1% NP40 and EDTA-free complete protease inhibitors) by tumbling for 10 min at 4°C. Cellular debris was removed by centrifugation at 10,000 *× g* for 10 min at 4°C. The Click-iT reaction was performed by incubating the supernatant with 1.9 mM CuSO_4_, 1.9 mg/ml ascorbic acid and 0.02 mM acetylene-PEG_4_-biotin (Jena Bioscience) for 30 min at room temperature. To remove unbound acetylene-PEG_4_-biotin, proteins were precipitated with 3 volumes of methanol, 0.75 volumes of chloroform and 2 volumes of H_2_O. After centrifugation at 21,000 × *g* for 2 min at room temperature, the liquid phase was removed, and the pellet was washed twice with 650 µl methanol. The pellet was then dried and dissolved in 120 µl resuspension buffer (1% SDS, 1% NP40 and EDTA-free complete protease inhibitors in PBS). Using this suspension, the amount of AHA incorporated into total protein was measured by Western blot analysis using streptavidin-HRP (Pierce, #21,130, 1:5000 dilution in 4% BSA-TBS-T).

For capture of AHA-labelled proteins, equal amounts of the suspension were transferred to low protein binding microcentrifuge tubes (Eppendorf) and the volume was adjusted to 900 µl with binding buffer (0.1% SDS, 1% NP40 and EDTA-free complete protease inhibitors in PBS). AHA-labelled proteins were then captured with 50 µl high capacity streptavidin resin (Thermo Fisher) at 4°C overnight. The beads were washed with 500 µl binding buffer under rotation for 15 min at room temperature. Washing was repeated twice with 4 M urea wash buffer (4 M urea and EDTA-free complete protease inhibitors in PBS), followed by two washes with 6 M urea wash buffer (6 M urea and EDTA-free complete protease inhibitors in PBS). Captured proteins were eluted by incubating the beads in 50 µl 2 × SDS sample buffer containing 1 mM biotin (Thermo Fisher) for 30 min at room temperature, followed by a heating step for 5 min at 95°C. The eluate was analysed by Western blotting using protein-specific antibodies.

## Supplementary Material

Supplemental Material

## Data Availability

The processed RNA-Seq and Ribo-Seq data have been deposited in the GEO database under the following accession number: GSE166783 (token for reviewer to access these data; wbqjeqkwpvejjyd). The mass spectrometry data have been deposited to the ProteomeXchange Consortium via the PRIDE partner repository with the data set identifier PXD029512 (login details for reviewers to access these data: Username: reviewer_pxd029512@ebi.ac.uk and Password: irhF9Jjb).
