# Supplementary material for: Preferential translation of p53 target genes
Source: RNA Biol. 2022 Apr 7;19(1):437–52. doi: 10.1080/15476286.2022.2048562 (PMC8993080; doi:10.1080/15476286.2022.2048562)

Figure 1, Hisaoka et al.

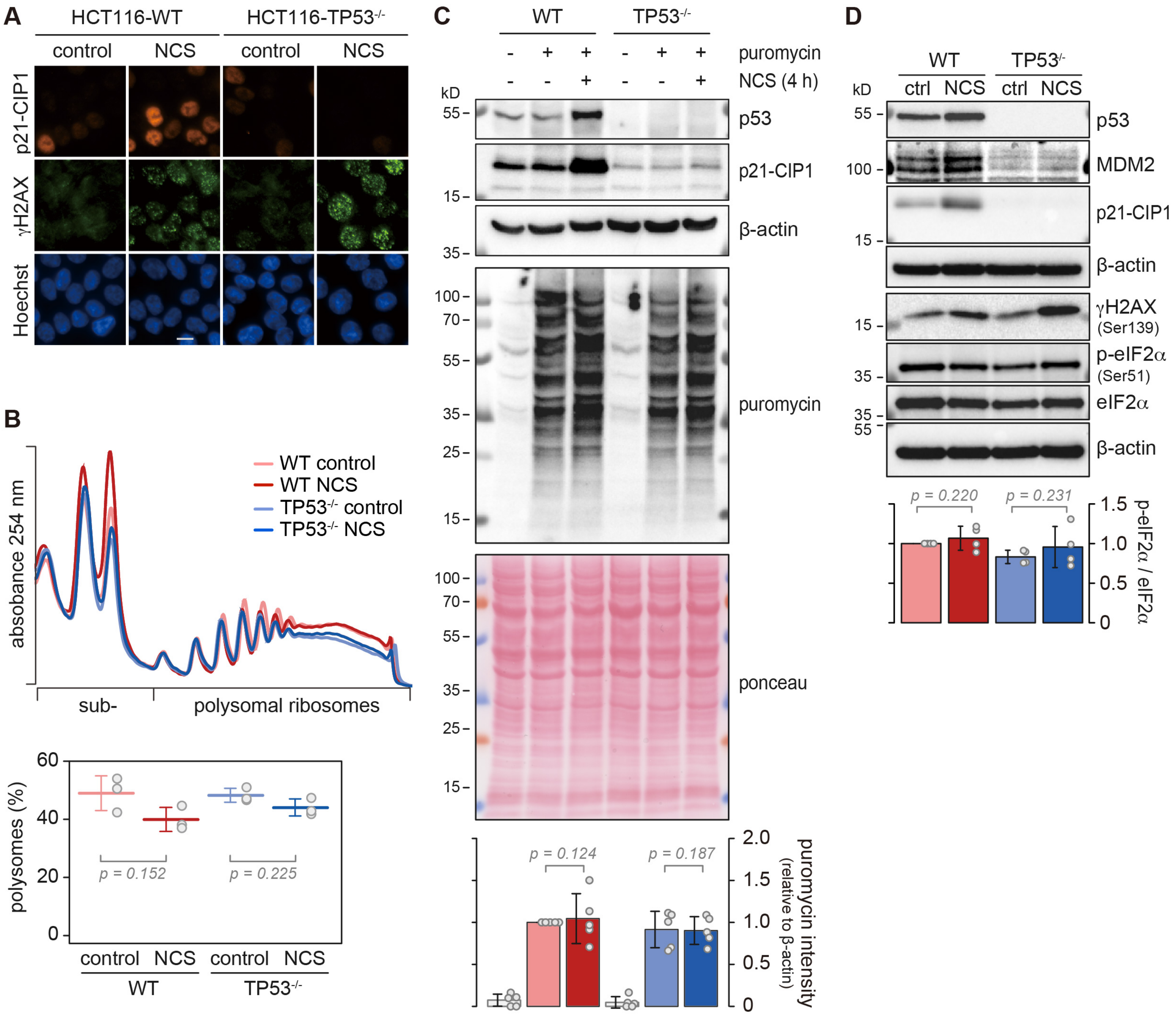

Figure 2, Hisaoka et al.

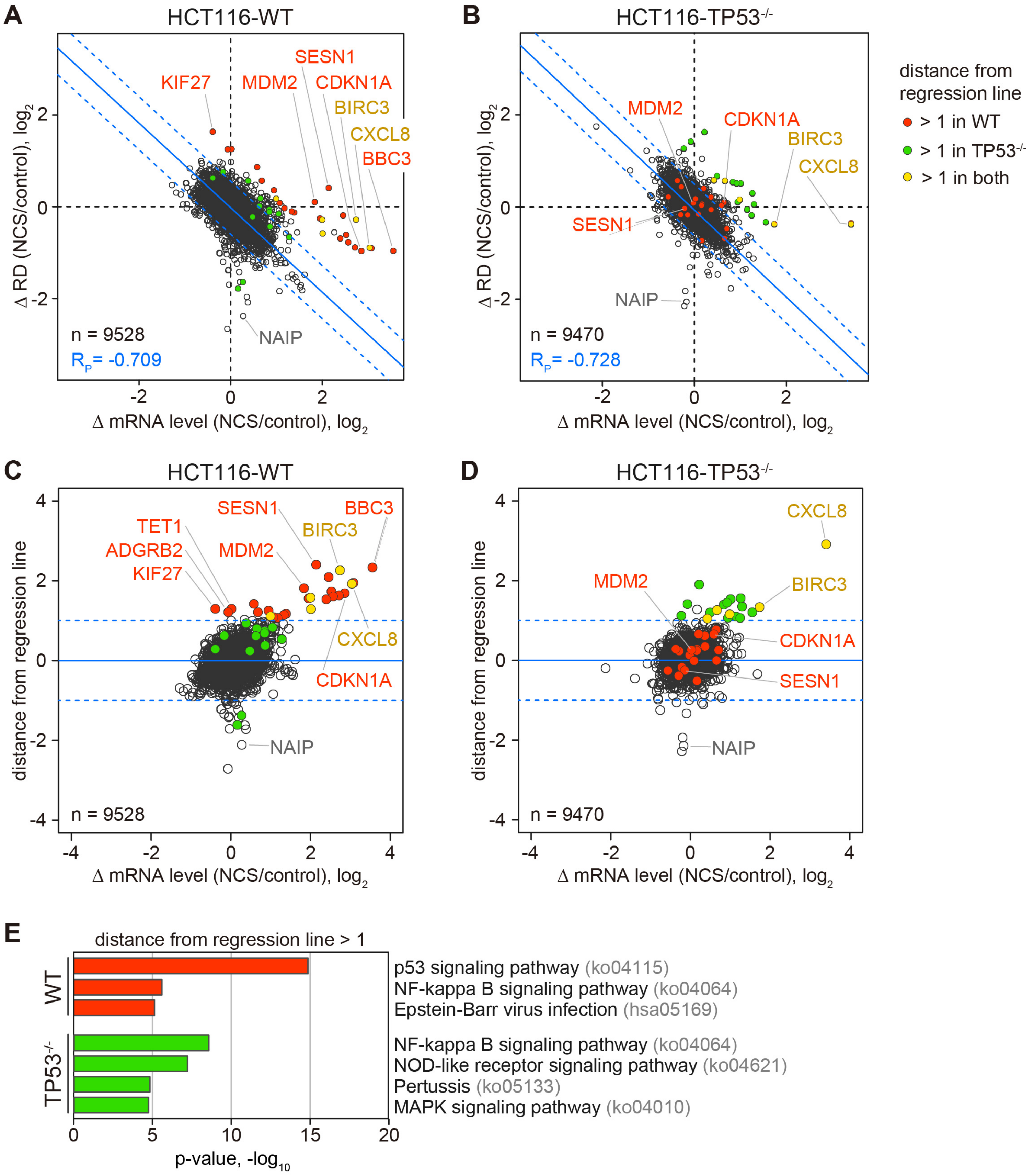

Figure 3, Hisaoka et al.

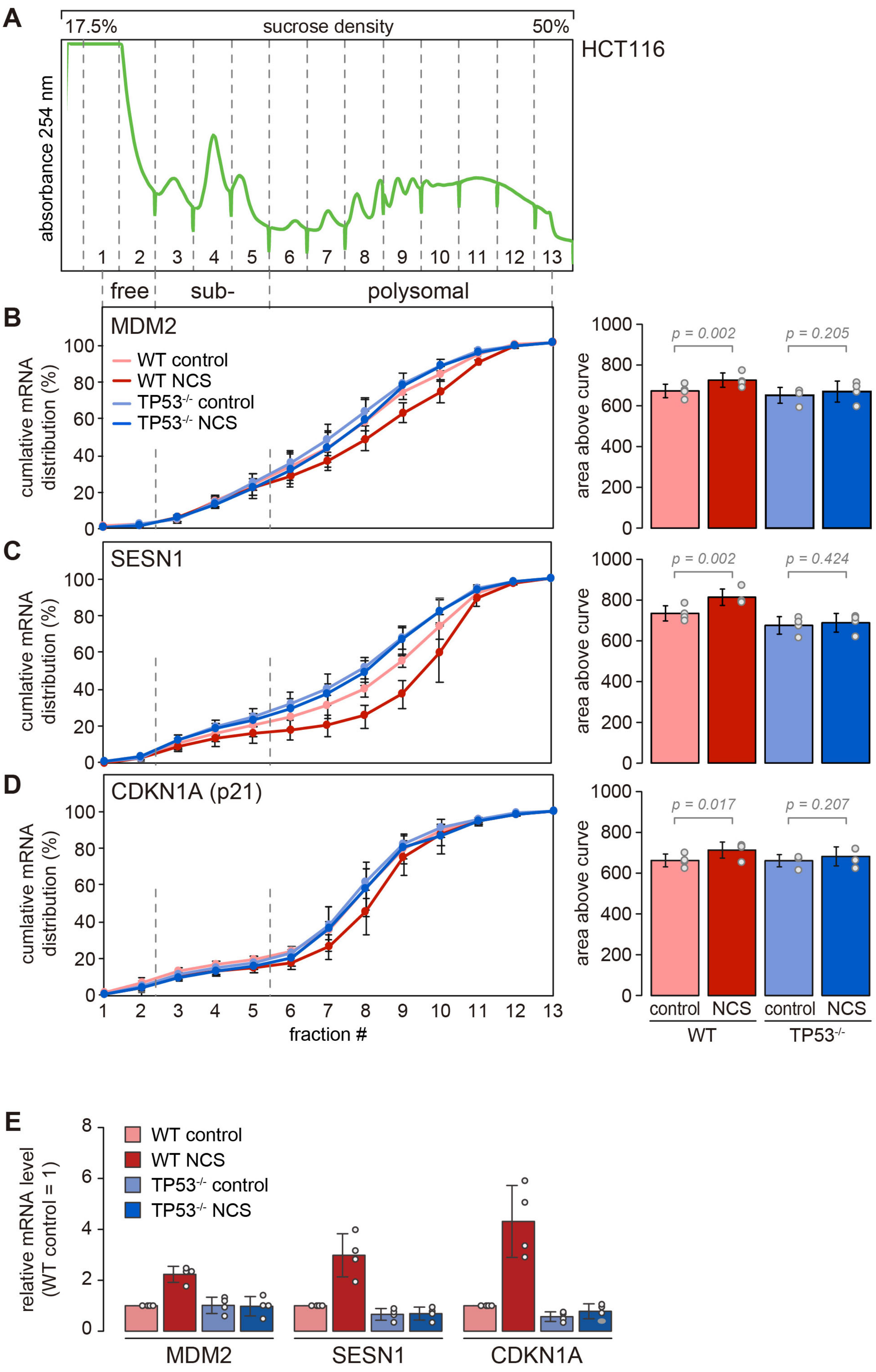

Figure 4, Hisaoka et al.

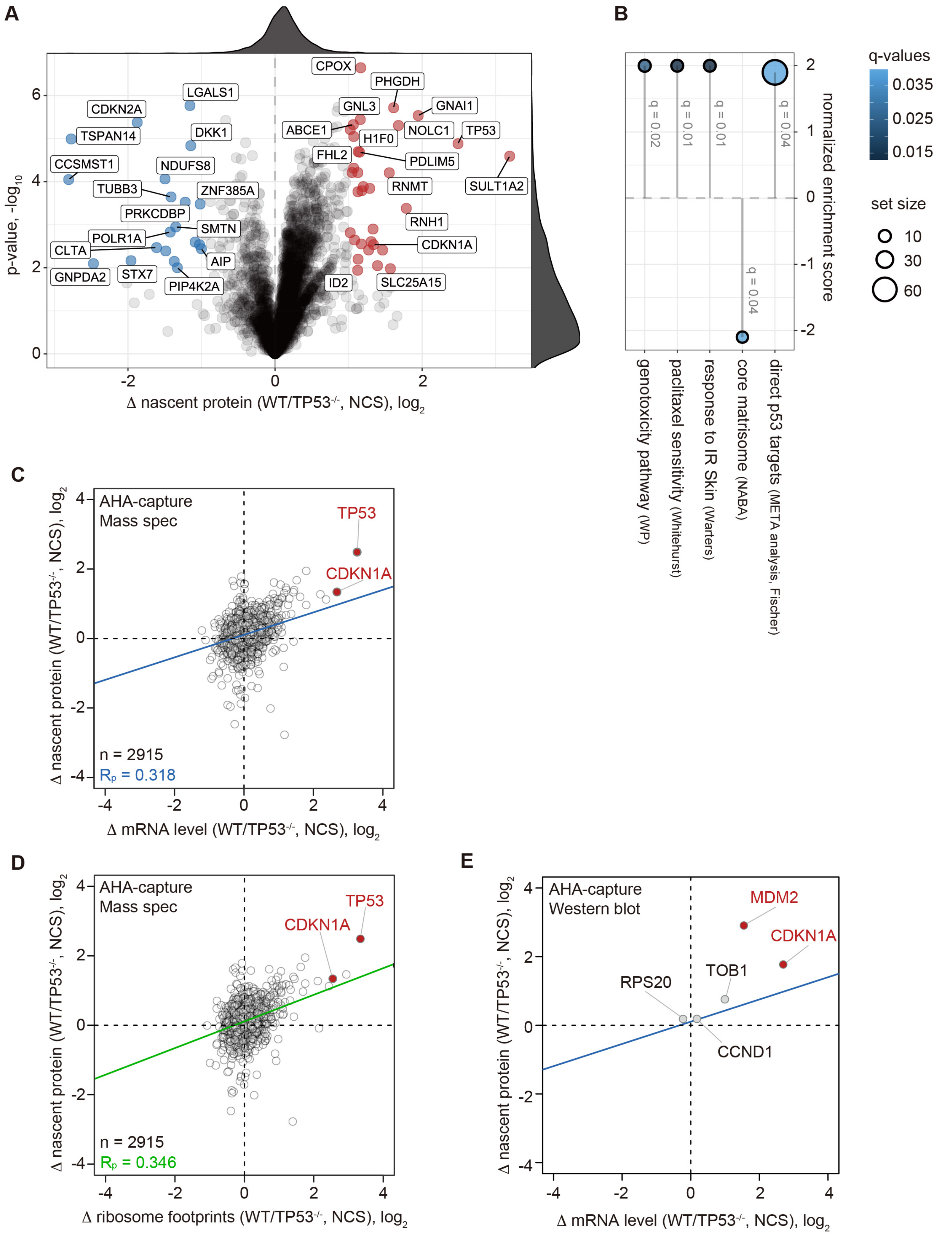

Figure 5, Hisaoka et al.

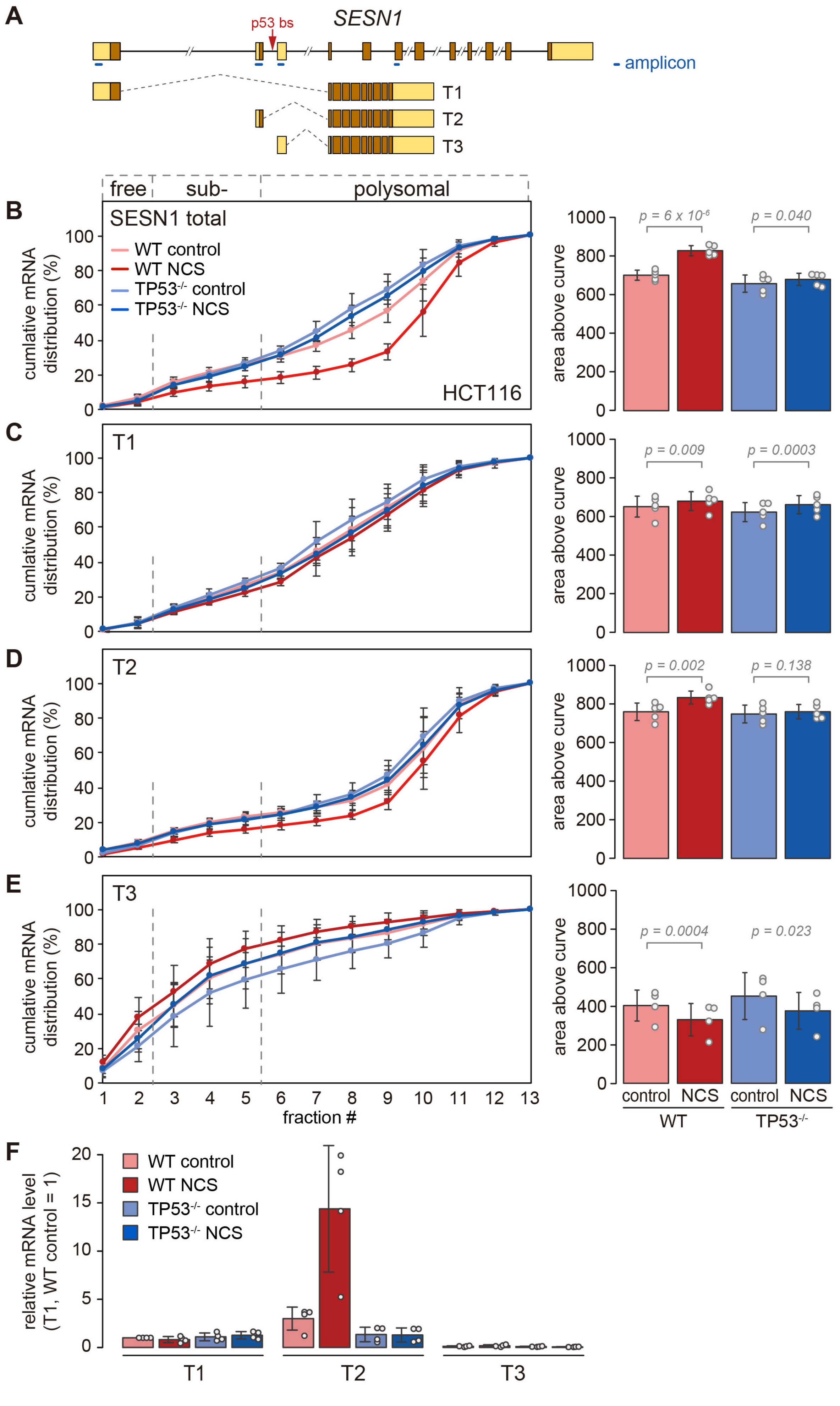

Figure 6, Hisaoka et al.

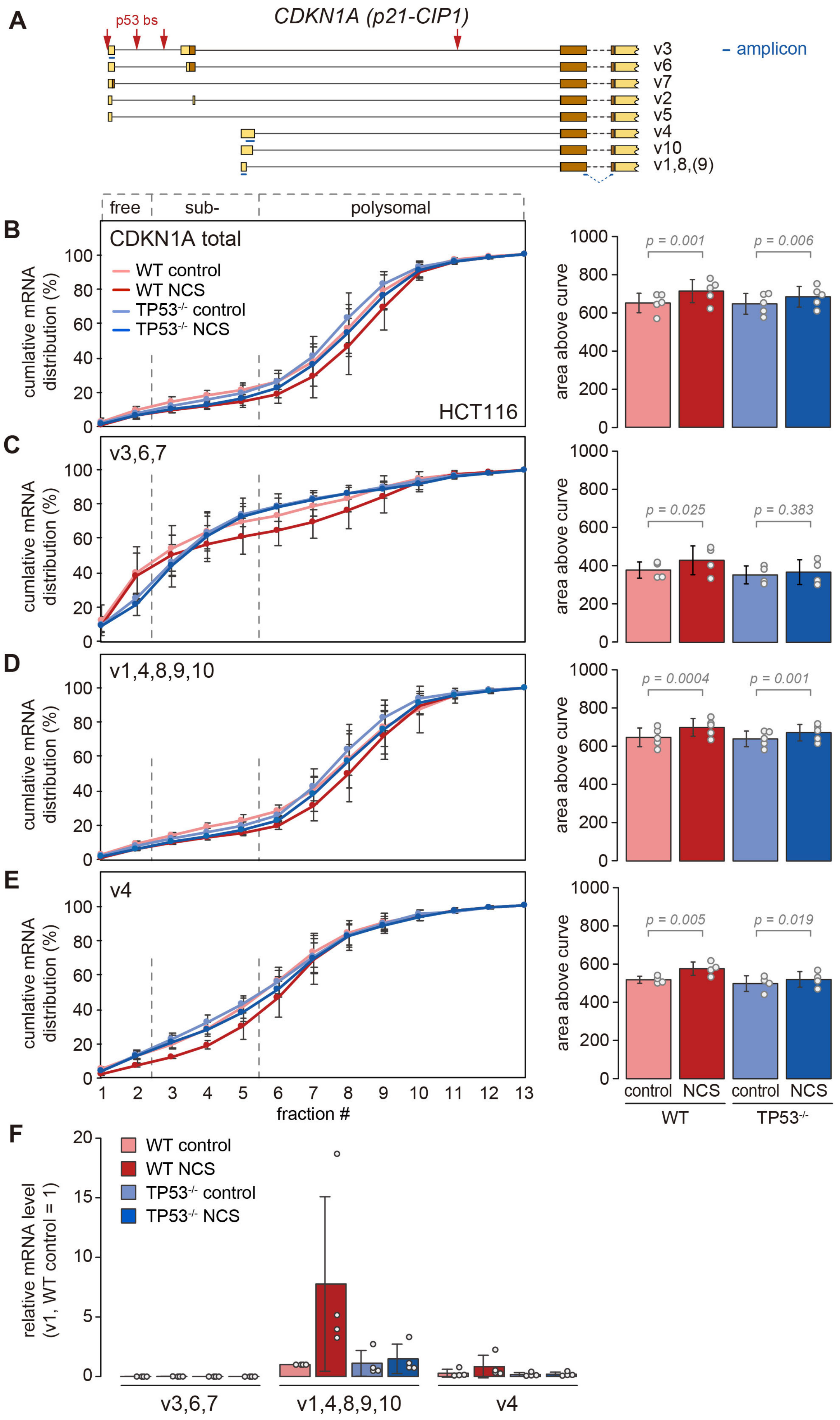

Supplement: Supplemental Material [file KRNB_A_2048562_SM7673.zip › downloadFromZipFile.pdf]
